# Antisense Oligonucleotide STK-002 Increases OPA1 in Retina and Improves Mitochondrial Function in Autosomal Dominant Optic Atrophy Cells

**DOI:** 10.1089/nat.2024.0022

**Published:** 2024-10-16

**Authors:** Aditya Venkatesh, Taylor McKenty, Syed Ali, Donna Sonntag, Shobha Ravipaty, Yanyan Cui, Deirdre Slate, Qian Lin, Anne Christiansen, Sarah Jacobson, Jacob Kach, Kian Huat Lim, Vaishnavi Srinivasan, Boris Zinshteyn, Isabel Aznarez, Laryssa A. Huryn, Zhiyu Li, Robert B. Hufnagel, Gene Liau, Karen Anderson, Jeff Hoger

**Affiliations:** ^1^ Stoke Therapeutics, Bedford, Massachusetts, USA.; ^2^ Ophthalmic Genetics and Visual Function Branch, National Eye Institute, National Institutes of Health, Bethesda, Maryland, USA.

**Keywords:** ADOA, OPA1, ASO, mRNA, exon splicing, haploinsufficiency

## Abstract

Autosomal dominant optic atrophy (ADOA) is an inherited optic neuropathy most frequently associated with *OPA1* mutations. Most variants result in haploinsufficiency, and patient cells express roughly half of the normal levels of OPA1 protein. OPA1 is a mitochondrial GTPase that is essential for normal mitochondrial function. We identified and characterized STK-002, an antisense oligonucleotide (ASO) designed to prevent the incorporation of a naturally occurring alternatively spliced nonproductive exon in *OPA1*. STK-002 dose dependently reduced the inclusion of this exon, and increased OPA1 protein in human cells, including ADOA patient-derived fibroblasts. ADOA patient cells manifest reduced mitochondrial respiration, and treatment with STK-002 improved the parameters of mitochondrial respiratory function in these cells. Since STK-002 increases OPA1 through the wild-type allele, we assessed retinal OPA1 in wild-type cynomolgus monkeys and rabbits after intravitreal administration of STK-002 or a rabbit-specific surrogate. Increased OPA1 protein was produced in retinal tissue in both species at 4 weeks after ASO injection and persisted in monkeys at 8 weeks. STK-002 and enhanced OPA1 immunofluorescence were visualized in retinal ganglion cells of cynomolgus monkeys treated with the ASO. Cumulatively, these data support the progression of STK-002 toward the clinic as the first potential disease-modifying treatment for ADOA.

## Introduction

Autosomal dominant optic atrophy (ADOA) is the most common form of inherited optic neuropathy, with a reported prevalence of ∼1:30,000 in the United Kingdom and worldwide^1^ and up to 1:10,000 in Denmark.^2^ ADOA can present clinically as an isolated bilateral optic neuropathy or as a complex phenotype called ADOA+ that includes extraocular signs.^3^ In ADOA the eyes are affected symmetrically with progressive loss of visual function that begins in childhood. The disease is often first detected as children enter school, or at the age of 5–7 years.^4,5^ Patients with ADOA display bilateral scotomas, or areas of impaired visual acuity, in their central visual field, with accompanying optic disc pallor caused by the loss of retinal ganglion cells (RGCs). Peripheral vision is typically spared.^1^ While complete loss of vision from ADOA is not likely, visual function can be severely impacted with up to 50% of affected individuals becoming legally blind.^3^ Patients with ADOA report reduced quality of life due to difficulties with common daily activities such as driving a car. Currently, there are no treatments for ADOA.

Variants in the nuclear-encoded *OPA1* gene have been identified in ∼70% of ADOA cases.^2,4,5^ To date, over 400 distinct and likely pathogenic variants have been identified.^5^ About 50% of *OPA1* mutations result in premature termination of translation, resulting in null alleles. As a result, OPA1 protein levels are substantially reduced in patient cells.^6^ Therefore, haploinsufficiency is believed to be the primary mechanism of disease underlying *OPA1*-associated ADOA. *OPA1* encodes a dynamin-related GTPase that localizes exclusively to the mitochondrial inner membrane and intermembranous space and is critical for normal mitochondrial function.^7^

Normal organization and structure of the mitochondrial cristae are dependent on OPA1.^8,9^ Additional roles for OPA1 have been described in calcium regulation, mitochondrial DNA (mtDNA) maintenance, and efficient function of the mitochondrial respiratory apparatus. OPA1 is also essential for successful mitochondrial fusion.^7,10,11^ Reduced OPA1, as documented in ADOA patient cells, has been shown to negatively impact these functions and to render cells more sensitive to apoptotic stimuli.^12,13^ RGCs, particularly those located in the central retina or fovea, appear to be highly sensitive to reduced levels of OPA1. Foveal RGCs are the cells affected in patients with *OPA1*-associated ADOA, and their dysfunction and/or loss accounts for the visual disturbances manifested with this disease.^3,14^

STK-002 is a synthetic antisense oligonucleotide (ASO) that has been designed to prevent the inclusion of a naturally occurring nonproductive alternatively spliced exon in the human *OPA1* pre-messenger RNA (pre-mRNA). Inclusion of this exon results in nonsense-mediated decay (NMD) of the transcript. STK-002 blocks the incorporation of this alternative exon to increase OPA1 protein levels. This approach capitalizes on the presence of one wild-type (WT) allele and is agnostic to the specific mutation on the other allele.^15^ In this study, we have demonstrated the potential therapeutic utility of STK-002 for ADOA with a series of studies intended to show the gene-specific and variant-independent nature of this approach and its potential effectiveness in the eye.

## Material and Methods

### Animal care

In-life phase of the rabbit and monkey studies were carried out by external contract research organizations (CROs) (Absorption Systems, USA [rabbit] and Alta Biosciences, USA [monkey]). All animal experiments were conducted in accordance with the National Research Council’s Guide for the Care and Use of Laboratory Animals under protocols approved by the Institutional Animal Care and Use Committee (IACUC) of Stoke Therapeutics and/or contracted facility.

### Bioinformatic analysis

Bioinformatic RNA-sequencing analysis of the Sequence Read archive with the identifiers SRP026048 (12 samples), SRP107937 (31 samples), SRP174668 (36 samples), and ERP003613 (4 samples) was performed by using the previously described bioinformatic tools, transcript database, and quality control of samples to identify NMD splicing events, exon inclusion and skipping, and alternative splice junctions and intron events.^15^

For analysis of nonproductive transcript levels in human retinal tissue, RNA-sequencing datasets ERP126780 (50 samples),^16^ SRP015336 (3 samples),^17^ and SRP107937 (16 samples)^18^ were used, while the SRP080000 dataset (11 retinal samples)^19^ was used for the monkey analysis. Transcripts per million (TPM) were obtained using Salmon v1.10.2, with a custom transcriptome, including unannotated splice events (identified as previously described^15^), and the level of nonproductive transcript inclusion was calculated as [nonproductive transcript TPM]/[total *OPA1* transcripts TPM] and expressed as a percentage. Conservation analysis of the NMD exon inclusion event on the *OPA1* gene across 100 different species was performed by using the human PhastCons 100-way conservation scores database, as previously described.^15^

### Cells and cell cultures

All cells used in the study were cultured at 37°C in a humidified incubator with 5% CO_2_. HEK293 cells were obtained from ATCC and cultured in DMEM high glucose with 10% FBS. HEK293 *OPA1^+/−^* cells were generated as a cellular model of haploinsufficiency with CRISPR technology at GenScript. Briefly, the *OPA1* gene was targeted and mutated by transient cotransfection of plasmids carrying gRNA and Cas9. Confirmation of the knockout was performed with Sanger sequencing followed by full-length sequencing of the *OPA1* gene. HEK293 cells that underwent the CRISPR process but maintained the *OPA1* WT sequence in both alleles were called *OPA1^+/+^* HEK293 cells.

Primary human dermal fibroblasts: WT primary dermal fibroblasts were purchased from ATCC (PCS-201-012) and the Coriell Institute (GM02036 and GM03234). The three human primary dermal fibroblasts from ADOA patients were obtained from the National Eye Institute. Fibroblast cells were grown in an enriched medium (1:1 basic fibroblast media and AmnioMax^TM^-II Complete medium) at 37°C in 5% CO_2_. The composition of the basic fibroblast media was as follows: DMEM high glucose with 10% heat-inactivated FBS, glutamine (2 mM final), sodium pyruvate (1 mM final), and antibiotics.

### Synthesis and chemistry of STK-002 and ST-1102

Both STK-002 and ST-1102 are uniform 2′O-methoxyethyl-modified oligonucleotides with a phosphorothioate backbone. STK-002 was either synthesized by IDT (for screening studies) or in-house (for *in vitro* characterization and *in vivo* studies), with sequence referenced in the patent WO2021231107A1.^20^ ST-1102 was synthesized in-house.

### Treatment with cycloheximide (CHX)

For any *in vitro* analysis of nonproductive transcript levels, cells were treated with their respective culture medium containing CHX (50 μg/mL) for 3 h immediately before harvesting. Control cells received culture medium containing an equivalent volume of DMSO.

### RNA extraction

For cultured cells, total RNA was extracted using the Qiagen RNeasy Kit (Qiagen, 74106) or RNeasy 96 Kit (Qiagen 74181) following the manufacturer’s instructions. RNA isolation from tissue was performed using the QIAzol reagent as described previously.^15^

### cDNA synthesis

cDNA synthesis was conducted using SuperScript™ IV Reverse Transcriptase (Thermo Fisher, 18090010) following the manufacturer’s instructions.

### ASO delivery: Transfection and gymnotic (free) uptake

For transfection, diluted Lipofectamine RNAiMAX reagent (0.9 μL in 100 μL OptiMem) was mixed with ASO at the indicated concentration in Eppendorf tubes for 30 min and placed in 24-well plates. Cells (100,000 cells, suspended in 400 μL media) were added to 24-well plates and incubated for 5–6 h at 37°C in 5% CO_2_. Media was then either replaced entirely or an additional 500 μL fresh media was added to wells and plates were returned to incubator until harvesting.

For gymnotic uptake, HEK293 cells (25,000–30,000 cells suspended in 450–490μL culture media) were added to 24-well plates containing concentrated ASO media (10–50μL) to obtain final desired ASO concentration. Cells were incubated for 72–96 h.

### RT-PCR and qPCR analysis

RT-PCR was performed as previously described^15^ with the primers and cycle conditions listed in Supplementary Tables S1 and S^2^. The intensities of the nonproductive and productive *OPA1* mRNA transcripts from the RT-PCR gel images were calculated using the Multi Gauge software (Fujifilm V2.3). Relative nonproductive transcript abundance was calculated as the intensity of the (nonproductive transcript/[the nonproductive + productive transcript] and expressed as a percentage.

The qPCR was also performed as previously described^15^ with primer-probe assay information and cycle conditions listed in the Supplementary Tables S3–S^5^. The 2–^ΔΔCt^ method was used to calculate relative expression levels. Total *OPA1* mRNA assessments in cell-based studies were normalized to housekeeping gene RPL32. Similarly, for EC50 evaluation, levels of nonproductive transcript were normalized to RPL32. For cynomolgus monkey studies, levels of nonproductive transcript were normalized to β-actin.

For all *in vitro* RT-PCR, qPCR, and protein studies, one biological replicate is defined as data obtained from an individual well of cells that received a specific treatment, as indicated in the experiment. For qPCR analysis, every biological replicate was run at least in duplicate, and these values were averaged to generate one data point.

### ATP measurement

*OPA1*^+/+^ or *OPA1*^+/−^ HEK293 cells were seeded at 3 × 10^5^ cells in a T-25 flask in cell culture media containing 10 µM STK-002 or an equivalent volume of Vehicle and incubated for 96 h. Every biological replicate in the data presented represents a separate flask of cells treated with STK-002 or Vehicle. Cells were washed twice with cold PBS and harvested with 120 mL cold ATP assay buffer. Samples were centrifuged at 13,000*g* for 5 min at 4°C and the supernatants were collected and kept on ice. An aliquot of lysed sample was set aside to be quantified by BCA assay for total protein concentration. The remaining sample was deproteinized using the Deproteinizing TCA Kit (Abcam, ab204708) and ATP levels were measured in the lysates using the ATP Assay Kit (Abcam, ab83355) according to the manufacturer’s instructions.

### *In vivo* assessment of ST-1102

Adult female New Zealand White (NZW) rabbits received a bilateral single IVT injection of 30 μL containing PBS (*n* = 3 per time point) or ST-1102 (0.04, 0.12, or 0.23 mg/eye). Clinical ophthalmic examinations were performed on days 8, 15, and 29. For nonproductive splicing and OPA1 protein analysis, each dose group included three animals for each time point. Retinas were divided along the nasal–temporal axes into two halves and collected in separate tubes, flash frozen, and stored below −70°C until analysis. The nasal halves were used to isolate RNA, whereas the temporal halves were used for protein analysis. ASO exposure was only measured on Day 29 and included three animals per dose group. Whole retinas were used for analysis.

### *In vivo* assessment of STK-002

Few 1.8- to 2.0-year-old male and female cynomolgus monkeys received a bilateral single 50 μL IVT injection of PBS (*n* = 4 animals per time point) or STK-002 (0.1, 0.3, and 1.0 mg/eye; *n* = 4 per dose) and followed for 4 weeks. A separate cohort at 0.3 and 1.0 mg/eye (*n* = 2 per dose) was followed for an additional 4 weeks. Clinical ophthalmic exams were performed at baseline and on study days 3, 15, 28 (cohorts 1 and 2), and 56 (cohort 2 only). Ocular tissues were harvested at week 4 or 8. One eye (OS) was dissected, retinal tissue was snap frozen, and the fellow eye (OD) was fixed intact in modified Davidson’s fixative, processed, and embedded in paraffin.

### Protein extraction and OPA1 protein assessment by western blot

Protein was extracted from rabbit retinal tissues in Red RINO tubes containing zirconium beads by adding RIPA buffer containing protease and phosphatase inhibitors (10 μL per mg tissue) followed by homogenization with a Bullet Blender Storm 24. Samples were then passed through the QIAshredder column by centrifugation. For protein extraction from cells, cell pellets were lysed in RIPA buffer and passed through the QIAshredder columns. Protein concentration in the flow through was measured by the BCA assay.

Immunoblotting was carried out as previously described^15^ with the following modifications. The following primary antibodies were used as follows: α-OPA1 protein (Abcam 119685), primary monoclonal antibody against β-actin (Abcam ab8226), and α-β-tubulin (Bio-Rad VMA00222). The secondary antibody used was the IRDye^®^ 800CW Donkey anti-mouse IgG secondary antibody (Licor, 926-32212). For detection, membranes were scanned on an Odyssey CLx Imaging System, and ImageJ or Multi Gauge software (Fujifilm V2.3) was used to quantify all OPA1 isoforms and β-actin or β-tubulin in each sample lane. OPA1 protein levels were normalized to β-actin or β-tubulin as indicated and expressed relative to the average OPA1 expression levels of the Vehicle group.

### OPA1 protein quantitation by ELISA

Cynomolgus monkey tissue homogenate was prepared by adding retina to prefilled tubes with zirconium beads (SPEX SamplePrep) at a 20 mg/mL concentration using 1X Lysis buffer (Ray Biotech) plus 100X protease and phosphatase Inhibitor cocktail (Thermo Fisher). Sample tubes were added to a precooled cryoblock and lysed in an SPEX 1600 Mini G homogenizer at 1425 rpm for 5 min. After homogenization, samples were centrifuged at 10,000*g* for 5 min at 4°C, and the supernatant was collected and transferred to a new polypropylene tube for analysis. The relative quantity of OPA1 protein in monkey retinal tissues was measured by an ELISA. Briefly, Nunc plates (Thermo Fisher) were coated with the captured mouse monoclonal antibody against OPA1 protein 500 ng/mL final concentration (BD Transduction Labs, 612007) overnight at 4°C. After the plates were washed, blocked with SuperBlock T20, (300 μL, Thermo Fisher) for 60 min at room temperature on a plate shaker and rinsed, the test samples, OPA1 calibration curve (Origene, 311417) and Vehicle (100 μL) were added to the plates and incubated for 2 h at room temperature on a plate shaker. After the plates were washed, the detection antibody HRP-rabbit monoclonal anti-OPA1 antibody, 1000 ng/mL final concentration, (Novus Biologicals) was added (100 μL), and plates were incubated for 60 min at room temperature in the dark on a plate shaker. After the plates were washed, the signal was developed with TMB substrate (Thermo Fisher) for 20 min and stopped in accordance with the manufacturer’s instructions. The plates were read at 450 nm by SpectraMax 5Me (Molecular Devices) and a four-parameter logistic model was used to generate the standard curve and the quantity of OPA1 in each test sample was extrapolated. OPA1 protein levels in each sample were normalized to the total protein content determined by the BCA assay.

### Cell viability assessment

Cell viability was assessed using the RealTime-Glo™ MT Cell Viability Assay from Promega (G9711) following the manufacturer’s instructions for the continuous-read method. Briefly, 5,000 HEK293 cells were plated in each well of a 96-well white, clear bottom plate in media containing ASO at the final desired concentration. Assay reagents were added at the same time and the plate was incubated at 37°C in a humidified incubator with 5% CO_2_. Luminescence was measured at 30 min and 72 h postplating. Luminescence readings at 30 min were subtracted from the 72-h readings to account for any initial differences in plating. Luminescence readings were normalized to Vehicle-treated wells to calculate relative changes in viability.

### Mitochondrial respiratory functions measured by Seahorse XFe96 analyzer

For characterization of mitochondrial bioenergetics in ADOA patient fibroblast cells, 10,000 fibroblast cells were plated in Seahorse XFe96 PDL plate for 24 h. The Seahorse XF Cell Mito Stress Test Kit (Agilent 103015–100) was used to assess mitochondrial function following the manufacturer’s instructions. The final concentration of the electron transport chain (ETC) modulators was: oligomycin (1.5 µM), FCCP (1 µM), and rotenone/antimycin (0.5 µM). Cell counts for each well were captured by staining with Cyquant and imaged using an ImageXpress (Molecular Devices). The Wave software program (Agilent Technologies V2.6.1) was used to normalize the oxygen consumption rate (OCR) to the cell count and reported as pmol/min/cell.

To study the effect of STK-002, fibroblast cells were treated with different doses of STK-002 by transfection using Lipofectamine RNAiMAX for 72 h. Cells were then trypsinized and replated on Seahorse XFe96 PDL plates for an additional 24 h, and the assay was performed as described above.

One biological replicate in an experiment represents an average of OCR values obtained from 8–12 wells of the XFe96 plate. Data plotted represent the mean ± standard deviation (SD) of three biological replicates.

### Quantification of ASO *in vivo* by hybridization enzyme linked immunosorbent assay (HELISA)

Before measuring ASO, retinal tissues were processed in tubes containing zirconium beads by adding homogenate buffer containing proteinase K (0.25 mg/mL) followed by homogenization with Spex 1600 MiniG homogenizer for 2 × 1 min at 1200 rpm. Then the homogenate was incubated at 55°C for an hour for tissue lysis. The concentrations of ST-1102 in rabbit and STK-002 in monkey retinal tissues were measured by using an established HELISA for quantifying ST-1102 or STK-002, respectively with the following modifications of previously described methods.^21,22^ Briefly, the complementary capture probe with biotin at 3′ end was coated onto a NeutrAvidin ELISA plate (Pierce 15507). Separately, the 5′ digoxigenin-labeled detection probe was incubated with a standard curve of ST-1102 samples or test samples. After the complex was hybridized with the capture probe, an anti-digoxigenin antibody conjugated to alkaline phosphatase (Sigma, 11093274910) was added to detect the bound analyte, and ATToPhos^®^ (Promega, S1000) was used as a substrate for fluorescent measurements with excitation wavelength of 435 nm and emission wavelength of 555 nm. The standard curves of ST-1102 and STK-002 were calculated with a four-parameter logistic, and the quantity in each test sample was extrapolated.

### *In situ* detection of STK-002 by miRNAscope™

Whole globes were harvested and fixed for 24 h in Modified Davidson’s fixative (MDF), then subjected to routine processing and paraffin embedding. A specific probe directed against the STK-002 sequence was generated and used for detection by custom miRNAscope LS RED *in situ* hybridization (probe dilution 1:5000). RNA *in situ* hybridization was performed on automation platform using the miRNAscope Red Reagent Kit (Advanced Cell Diagnostics, Inc., Newark, CA) according to the manufacturer’s instructions. Briefly, 5 μm MDF-fixed, paraffin-embedded tissue sections were pretreated with heat and protease before hybridization with the target oligo probes. Preamplifier, amplifier, and AP-labeled oligos were then hybridized sequentially, followed by chromogenic precipitate development. Each sample was quality controlled for RNA integrity with miRNAscope probe specific to PPIB RNA and for background with a probe specific to bacterial dapB RNA. Specific RNA staining signal was identified as red, punctate dots. Samples were counterstained with Gill’s Hematoxylin. Regions of interest (ROI) included RGC layer in or near the fovea and RGC layer in the peripheral retina. Visual scoring was performed by a qualified scientist who was blinded to treatment groups but not controls. The miRNAscope quantification is based on number of dots per cell and not on intensity. Percentage of cells positive is scored visually based on the number of cells with ≥1 dot/cell. Quantitative image analysis was performed using HALO^®^ software on 1 mm lengths of each ROI.

### Immunofluorescence detection of OPA1 protein

Eyes were fixed and embedded as described above. Immunostaining was performed on Leica automation platforms using the Bond™ Polymer Refine Detection Kit (Leica Biosystems). Briefly, 5 μm MDF-fixed tissue sections were pretreated with heat and target retrieval solution before incubation with the target antibody (Novus Anti-OPA1 antibody NB110‐55290 at 5 μg/mL), and polymer. Fluorescent whole slide images were acquired using 3DHistech Panoramic SCAN II digital slide scanner equipped with a 40x objective. Images were obtained from the regions of interest as described above. Quantitative image analysis was performed using HALO software on 1 mm lengths of each ROI.

### Biostatistical analysis

Differences among groups were determined by ordinary one-way analysis of variance (ANOVA) followed by Dunnett’s multiple comparison test or by unpaired *t*-test, as indicated. All statistical comparisons were performed with GraphPad Prism. Levels of significance began at *P* = 0.05.

## Results

### Validation of the nonproductive exon inclusion splicing event in *OPA1*

**FIG. 1.**
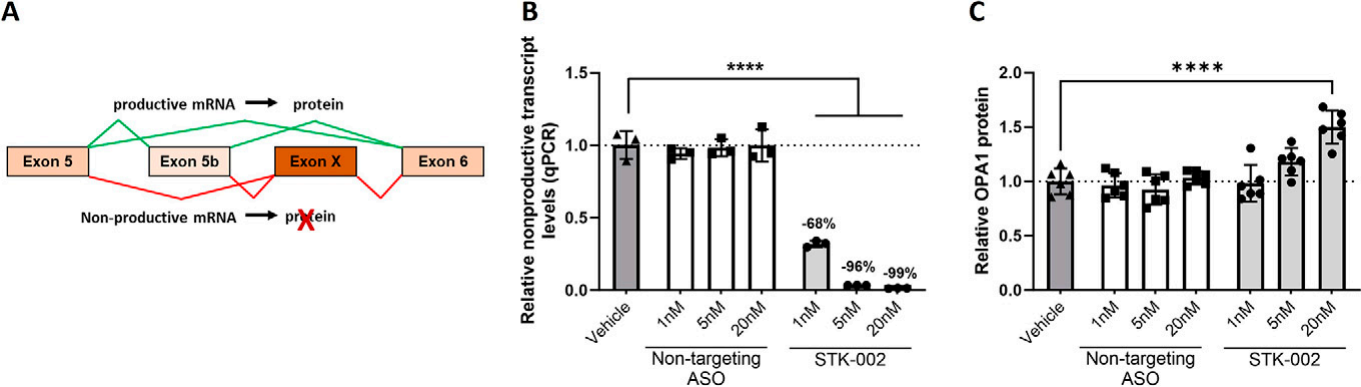
STK-002 reduces nonproductive transcript inclusion and increases OPA1 protein levels in HEK293 cells. **(A)** The inclusion of an alternative exon (Exon X) in the *OPA1* gene results in a nonproductive mRNA transcript, which is degraded by NMD. Exon X can occur downstream of either exon 5 or exon 5b, due to alternative splicing of exon 5b. **(B)** and **(C)** HEK293 cells were transfected with Vehicle or 1, 5, or 20 nM doses of STK-002 or nontargeting (NT) ASO. For nonproductive transcript analysis, cells were treated with CHX-containing media for 3 h before RNA isolation. RNA was isolated 24 h posttransfection and nonproductive transcript abundance was measured by qPCR and normalized to housekeeping gene *RPL32* **(B)**. For protein analysis, cells were lysed with RIPA buffer 72 h after transfection and western blots were probed with antibodies targeting OPA1 and β-tubulin **(C)**. Western blot images are in Supplementary Fig. S3. Data in **(B)** and **(C)** are plotted relative to the Vehicle group. RNA and protein data represent mean ± SD of three and six biological replicates, respectively. ****P < 0.0001 by ordinary one-way ANOVA followed by Dunnett’s multiple comparisons test. ANOVA, analysis of variance; NMD, nonsense-mediated decay; SD, standard deviation.

A novel alternative exon (chr3:193628509-193628616) was identified in the human *OPA1* gene by bioinformatics analysis, as previously described.^15^ This alternative exon contains a premature termination codon, and its inclusion should result in a nonproductive mRNA transcript that is degraded by NMD during translation. This alternatively spliced NMD exon, henceforth called Exon X, is present either between exons 5 and 6 (5-X-6) or between exons 5b and 6 (5-5b-X-6) in the *OPA1* gene, due to alternative splicing of exon 5b (Fig. 1A). RT-PCR assays designed to amplify exons 5, 5b, and 6 detected the presence of the nonproductive transcript in HEK293 cells and an increase in nonproductive transcripts was observed upon treatment with the translation inhibitor cycloheximide (CHX), confirming that the nonproductive transcript is degraded by NMD (Supplementary Fig. S1). Given the complex alternative splicing in this region, we performed RNA-seq on HEK293 cells treated with or without CHX to accurately quantify the relative abundance of nonproductive transcript relative to total *OPA1* transcripts. In HEK293 cells, our analysis showed that nonproductive transcripts accounted for 1.7% of total *OPA1* transcripts in the absence of CHX, which increased to 7.6% upon NMD inhibition with CHX. To test whether the nonproductive transcript was also present in human retinal tissue, we analyzed three publicly available RNA-sequencing datasets (Supplementary Fig. S2). In the 37 human retinal samples analyzed, the relative abundance of the nonproductive transcript ranged from 0.94% to 10.4% of total *OPA1* transcripts, with an average abundance of 3.8%. However, given that NMD is active in retinal tissue and the transcript is being continuously degraded, the true abundance of the transcript is potentially higher than these numbers.

### STK-002 decreases nonproductive OPA1 mRNA splicing and increases OPA1 protein *in vitro*

Targeted augmentation of nuclear gene output (TANGO) is a therapeutic approach that uses ASOs to target nonproductive splicing events and increase protein expression.^15^ To test whether *OPA1* is amenable to TANGO, we designed a series of ASOs that bind to Exon X and 100 bp into the surrounding intronic regions. All ASOs were based on 2′-methoxyethyl modification of the oligonucleotide with a phosphorothioate backbone. STK-002 was identified as the most potent ASO from these screening studies and was further evaluated. To characterize the effect of STK-002, we used a primer-probe-based qPCR assay that spans Exons X and Exon 6 to quantitatively measure the reduction of nonproductive transcript levels. Since Exon 6 is present in all *OPA1* isoforms, this qPCR assay would capture all transcripts containing Exon X. Transfection of HEK293 cells with STK-002 showed a dose-related reduction in nonproductive transcripts by qPCR (Fig. 1B). The reduction of nonproductive mRNA led to an increase in OPA1 protein (Fig. 1C, Supplementary Fig. S3) as measured by western blot. A nontargeting (NT) ASO control did not have any effect on either parameter, which demonstrates the specificity of the observed effects. (Fig. 1B, 1C). The increase in OPA1 protein upon STK-002 treatment exceeded the level of nonproductive transcript levels in HEK293 cells, which suggests that there may be posttranscriptional effects that contribute to the increased OPA1 protein levels upon STK-002 treatment. To calculate the half maximal effective concentration (EC50) in HEK293 cells, STK-002 was delivered by gymnotic uptake, and levels of the nonproductive transcript were detected by qPCR. A dose-dependent reduction in nonproductive transcripts was seen by gymnotic uptake and the EC50 for STK-002 was determined as 5.63 μM. (Supplementary Fig. S4). No effects on cell viability were observed at any of these doses with STK-002 treatment. (Supplementary Fig. S5).

### STK-002 increases total cellular ATP levels in *OPA1^+/−^* HEK293 cells

**FIG. 2.**
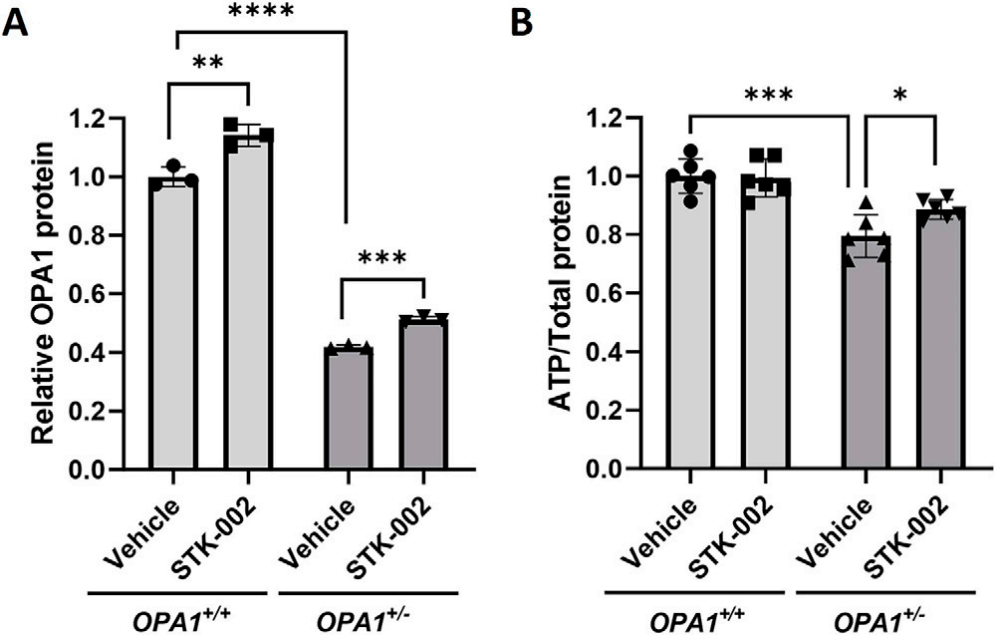
STK-002 increases OPA1 protein and total ATP levels in *OPA1^+/−^* HEK293 cells. **(A)** *OPA1^+/+^* and *OPA1^+/−^* HEK293 cells were treated with Vehicle or 10 µM STK-002 by gymnotic uptake and assessed for effect on OPA1 protein by western blot 72 h posttreatment (western blot images in Supplementary Fig. S6). Protein data are normalized to β-actin and expressed relative to the Vehicle group in *OPA1^+/+^* cells. The mean ± SD of three biological replicates is shown for each cell and treatment. **(B)** *OPA1^+/+^* and *OPA1^+/−^* HEK293 cells were treated with Vehicle or 10 µM STK-002 by gymnotic uptake for 96 h and total cellular ATP levels were measured and normalized to total protein levels. All values were plotted relative to the mean ATP level of *OPA1^+/+^* cells. The mean ± SD of six biological replicates is shown. *P < 0.05, **P < 0.01, ***P < 0.001, and ****P < 0.0001 by unpaired *t*-test.

Since most patients with ADOA show haploinsufficiency of *OPA1*, a cellular model of *OPA1* haploinsufficiency was engineered in HEK293 cells by using CRISPR-Cas9 gene editing to insert one nucleotide in one *OPA1* allele. The process yielded an *OPA1*^+/−^ HEK293 cell line that showed reduced OPA1 expression compared with an isogenic control cell line (*OPA1*^+/+^ HEK293) that underwent the gene editing process but did not develop any mutations (Fig. 2A, compares Vehicle groups of *OPA1*^+/+^ and *OPA1*^+/−^ cells). STK-002 increased OPA1 protein in both *OPA1*^+/+^ and *OPA1*^+/−^ HEK293 cell lines by gymnotic uptake (Fig. 2A, Supplementary Fig. S6).

Mitochondria are primary centers of ATP production in the cell through oxidative phosphorylation. Given the critical role of OPA1 in the maintenance of mitochondria bioenergetics, we investigated whether total cellular ATP levels were reduced in *OPA1*^+/−^ HEK293 cells. We found that total cellular ATP levels were significantly lower in *OPA1*^+/−^ HEK293 cells compared with isogenic WT control cells (Fig. 2B). STK-002 treatment increased ATP levels in *OPA1*^+/−^ HEK293 cells but did not modulate ATP levels in *OPA1*^+/+^ cells (Fig. 2B). These results support the concept that STK-002 can positively modulate the reduced ATP phenotype found in *OPA1*^+/−^ cells.

### STK-002 increases OPA1 expression in ADOA patient fibroblast cells in a variant-independent manner

**FIG. 3.**
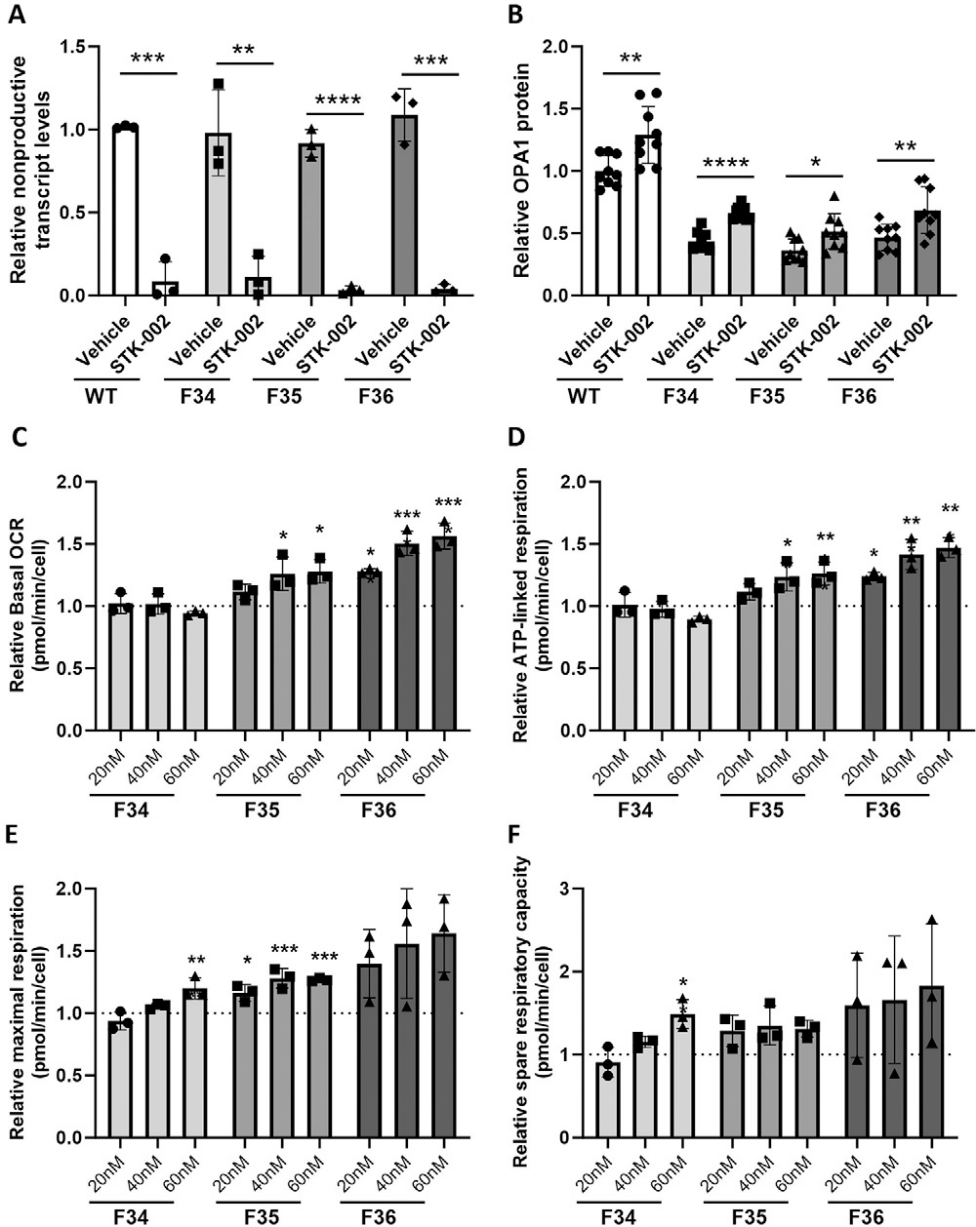
STK-002 reduces nonproductive exon inclusion, increases OPA1 expression, and mitochondrial function in ADOA patient fibroblast cells. WT (ATCC fibroblasts) and all three ADOA patient fibroblast cells were treated with 40 nM STK-002 by transfection and harvested after 24 h for analysis of nonproductive splicing **(A)** normalized to *RPL32*. OPA1 protein levels **(B)** were determined by western blot 72 h after transfection and normalized to β-actin (western blot images in Supplementary Fig. S8). qPCR data show mean ± SD of three biological replicates, while protein data show mean ± SD of nine biological replicates. Data are plotted relative to the WT Vehicle group. *P < 0.05, **P < 0.01, ***P < 0.001, and ****P < 0.0001 by unpaired *t*-test. **(C–E)** F34, F35, and F36 patient fibroblast cells were treated with 20, 40, or 60 nM STK-002 or Vehicle by transfection and their oxygen consumption rate (OCR; pmol/min/cell) was measured on a Seahorse XFe96 extracellular flux analyzer under basal and stress conditions and normalized to cell count. Basal respiration **(C)**, ATP-linked respiration **(D)**, maximal respiration **(E)**, and spare respiratory capacity **(F)** were reported. Data show mean ± SD of three biological replicates and plotted relative to the Vehicle group for each cell type. Vehicle group bars are not shown as it is 1.0 for each cell type. Each data point represents an average of OCR values from 8 to 12 wells. Statistical analysis was by ordinary one-way ANOVA followed by Dunnett’s multiple comparison test against Vehicle for each cell type. *P < 0.05; **P < 0.01; ***P < 0.001; and ****P < 0.0001. ADOA, autosomal dominant optic atrophy; WT, wild type.

Previous studies have used primary dermal fibroblasts derived from patients with ADOA *OPA1* mutations as an *in vitro* model of disease pathophysiology.^6,8^ To test whether STK-002 would increase OPA1 expression and potentially modify disease phenotype in ADOA, we evaluated dermal fibroblasts from three unrelated ADOA patients with different *OPA1* pathogenic variants (Supplementary Fig. S7 A). All three patient fibroblast cells showed reduced *OPA1* mRNA and OPA1 protein levels compared with three different WT fibroblast cells (Supplementary Fig. S7 B–D). Treatment with STK-002 reduced nonproductive splicing and increased OPA1 protein in WT fibroblasts as well as all three patient cells (Fig. 3A, B, Supplementary Fig. S8), supporting the mutation-independent nature of the TANGO approach.

### STK-002 enhances the ability of ADOA patient fibroblast cells to respond to mitochondrial stress

The respiratory function of the three patient cells was compared with all three WT fibroblast cells by measuring the OCR of live cells under basal conditions and upon treatment with different ETC inhibitors using the Seahorse extracellular flux analyzer. Specifically, basal respiration, ATP-linked respiration, maximal respiration, and spare (reserve) mitochondrial respiratory capacity were measured (Supplementary Fig. S9). All three ADOA patient cells showed a deficit in basal and ATP-linked respiration compared with the three WT cells. Maximal respiration and spare respiratory capacity were reduced in the three patient cells compared with WT cells from ATCC, while the other two WT cells showed more variability in these two parameters. This variability among the WT fibroblast cells could be attributed to differences in age, sex, and genetic backgrounds of the three donors, as well as methods of isolation and generation of cell lines, and therefore we cannot establish a baseline for mitochondrial bioenergetics in *OPA1^+/+^* fibroblasts.

We next evaluated whether treatment with STK-002 enhanced the parameters of mitochondrial bioenergetics in the three ADOA patient cells (Fig. 3C–F). STK-002 increased maximal respiration and spare respiratory capacity in all three patient fibroblast cells. STK-002 also increased basal OCR and ATP-linked respiration in patient cells, F35 and F36. A nontargeting ASO was tested in F35 cells and no change in any of the parameters was observed, suggesting that the increases observed in mitochondrial respiratory parameters were specific to STK-002 (Supplementary Fig. S10). Maximal respiration is measured upon the addition of the uncoupler FCCP, which mimics a physiological energy demand by stimulating the respiratory chain to operate at maximum capacity. The difference between maximal and basal respiration is the spare respiratory capacity, a measure of mitochondrial reserve. The increase in maximal respiration and spare respiratory capacity in all three patient cells with different pathogenic variants shows that STK-002 enhances the ability of *OPA1* haploinsufficient cells to respond to conditions that induce mitochondrial stress.

### Surrogate ASO ST-1102 produces lasting reduction in nonproductive splicing and increases OPA1 protein in rabbit retinas

**FIG. 4.**
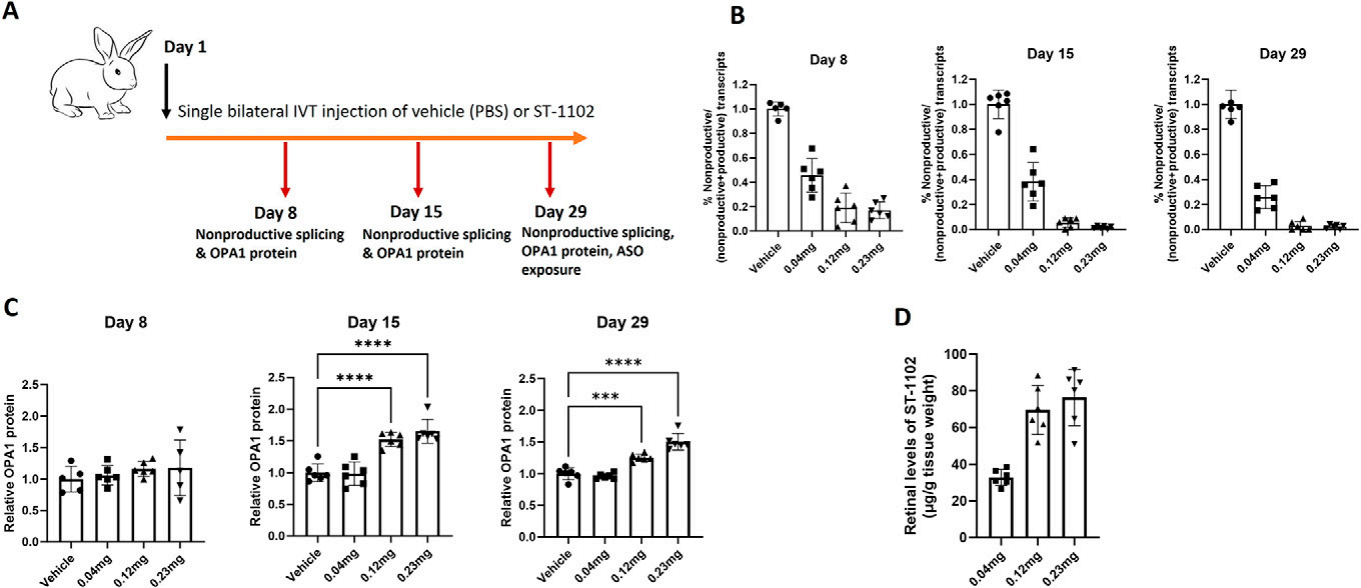
Surrogate ASO ST-1102 increases OPA1 protein in WT rabbit retinal tissue following single intravitreal (IVT) injection. **(A)** Adult Female New Zealand white rabbits were administered with a single, bilateral IVT injection of Vehicle (PBS) or 0.04, 0.12, or 0.23 mg/eye doses of ST-1102. Retinas were harvested 7, 14, or 28 days after injection for analysis of target engagement, OPA1 protein, and ASO exposure. **(B)** The effect on *OPA1* nonproductive transcripts in retina was measured by RT-PCR by quantifying the abundance of nonproductive *OPA1* mRNA transcripts (Ex5b-X-6) to the sum of productive (Ex5b-6) and nonproductive (Ex5b-X-6) transcripts and expressed as a percentage. RT-PCR gel images are included in Supplementary Fig. S11. Statistical significance (*P* < 0.0001 vs. Vehicle injected group) was observed at all time points for all dose groups. **(C)** OPA1 protein was quantitated in retinal lysates by western blot and normalized to actin (***P < 0.001, ****P < 0.0001 vs. Vehicle group). Western blot images are included in Supplementary Fig. S12. Data are plotted relative to the Vehicle group. **(D)** ST-1102 was detected in retinal tissue on Day 29 by HELISA at all three dose levels. Statistical analysis by ordinary one-way ANOVA followed by Dunnett’s multiple comparisons test.

Sequence alignments of the nonproductive exon suggested that it is well conserved in rabbit, pig, dog, and several nonhuman primate species (chimpanzee, rhesus monkey, green monkey). Interestingly, neither mouse nor rat have the nonproductive exon due to lack of conservation at the 3′ splice site and therefore, rodents could not be used as models for *in vivo* proof-of-mechanism studies. While the event is conserved in rabbits, the STK-002 target region is not perfectly conserved between humans and rabbits. Therefore, a rabbit surrogate ASO, ST-1102, with identical sequence complementarity to the rabbit *OPA1* target region was created for *in vivo* proof-of-mechanism studies. A single, bilateral intravitreal (IVT) injection of Vehicle (PBS) or ST-1102 at 0.04, 0.12, or 0.23 mg/eye was administered to female adult NZW rabbits and retinas were collected 7-, 14-, and 28-day postinjection for pharmacological assessments (Fig. 4A).

A significant dose-related decrease in the nonproductive transcripts was observed in rabbit retinal tissue as early as 7 days postinjection by RT-PCR. The levels of nonproductive transcripts further dropped at both 14- and 28-day postinjection (Fig. 4B and Supplementary Fig. S11). This analysis is specific to the 5b-X-6 nonproductive transcript as the qPCR assay that captures all nonproductive transcript was not available at the time of this experiment. No changes in retinal OPA1 protein levels were observed 7 days postinjection; however, significant dose-related upregulation of OPA1 protein levels was seen 14 days postinjection that remained significantly elevated at 28 days postinjection (Fig. 4C and Supplementary Fig. S12). Retinal exposure of ST-1102 was measured only 28-day postinjection. ST-1102 retinal levels increased with dose but were not strictly dose proportionate (Fig. 4D). Taken together, these data indicate that a single IVT administration of ST-1102 significantly reduced the levels of nonproductive transcripts and increased OPA1 protein in the rabbit retina for at least four weeks. Clinical ophthalmic exams showed that the ASO was well tolerated at all dose levels tested.

### STK-002 produces lasting reduction in NMD exon inclusion, increase in OPA1 protein, and exposure in cynomolgus monkey retinal tissue

**FIG. 5.**
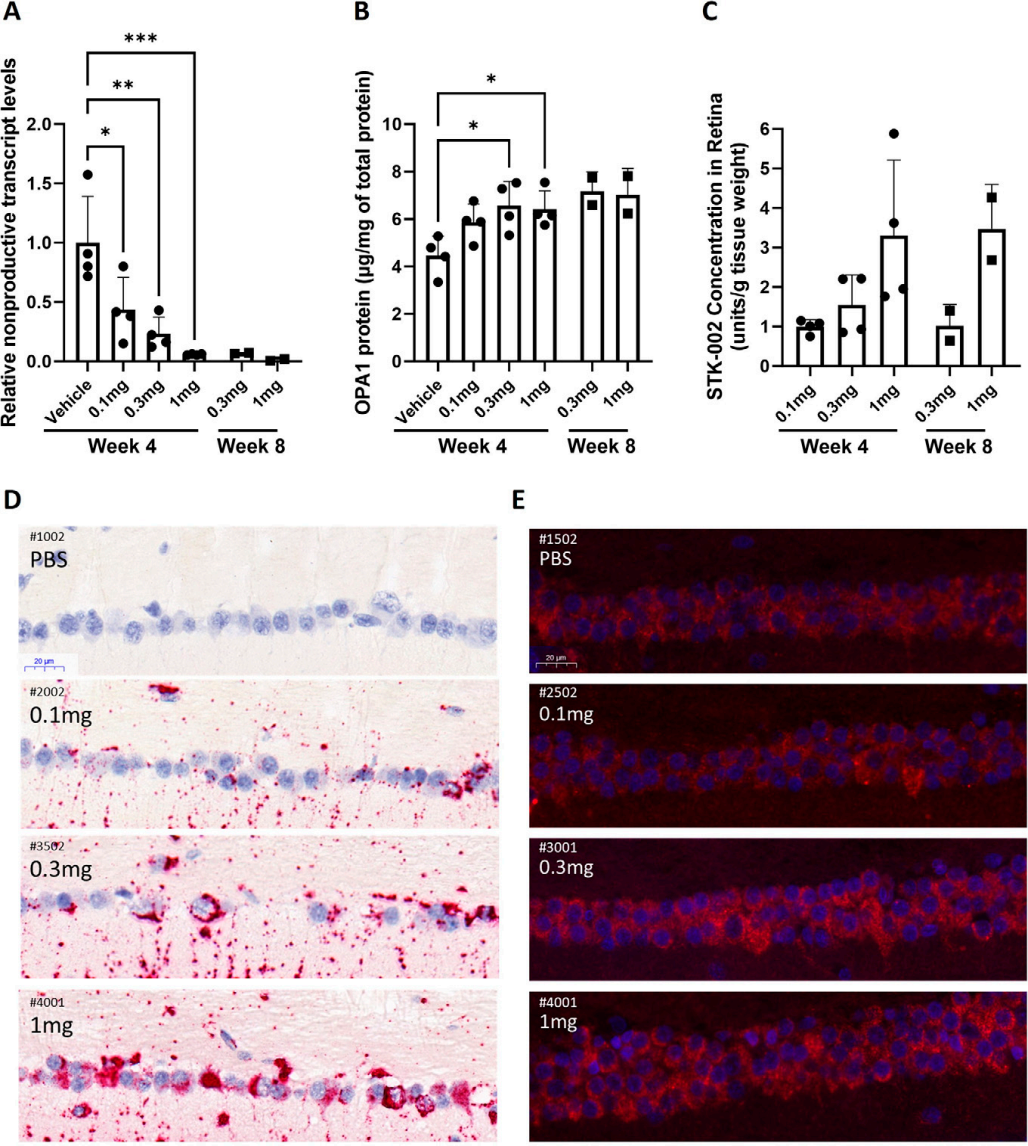
Intravitreal administration of STK-002 induces dose-related reduction in NMD exon inclusion and increased OPA1 protein with sustained tissue exposure in the retina of cynomolgus monkeys. Retinas were collected from indicated treatment groups at 4 or 8 weeks **(A)** The presence of *OPA1* nonproductive transcripts in retinal tissue was assessed by qPCR normalized to β-actin and expressed relative to the Vehicle group. **(B)** OPA1 protein was quantitated in retinal lysates by ELISA and normalized to total protein in the sample. For **(A)** and **(B)**, statistical analysis by ordinary one-way ANOVA followed by Dunnett’s multiple comparisons test. *P < 0.05, **P < 0.01; ***P < 0.001 vs. Vehicle group. Statistics were not performed for week 8 samples where *n* = 2. **(C)** STK-002 level in retinal tissue was quantitated by HELISA. Samples were normalized by tissue weight and plotted relative to the 0.1 mg group. Data are presented as mean ± SD. Each data point represents one retina. **(D, E)** Detection of STK-002 and OPA1 in retinal ganglion cells (RGCs). Sample images are presented and treatment type and STK-002 dose per eye are indicated. **(D)** miRNAscope™ staining with a specific probe to STK-002 was performed on MDF-fixed, paraffin-embedded cynomolgus monkey eyes to demonstrate the location of STK-002 (red) in RGCs. Increased STK-002 signal is correlated with increased dose. Nuclei were counterstained with hematoxylin (blue). **(E)** Immunofluorescence staining with OPA1 antibody shows an apparent protein increase (red) at 0.3 and 1.0 mg in the RGCs in/near the fovea. Nuclei stained with DAPI (blue). Scale bars: 20 µm.

As we demonstrated pharmacology in rabbits with a surrogate ASO, we set to investigate STK-002 pharmacology, exposure, and tolerability in cynomolgus monkeys. The STK-002 ASO target sequence is perfectly conserved, and the presence of the nonproductive transcript was confirmed in cynomolgus monkey retinal tissue (Supplementary Fig. S13). A single, bilateral IVT injection of Vehicle (PBS) or STK-002 was administered to cynomolgus monkeys. Pharmacology was assessed in the Vehicle, and 0.1, 0.3, and 1.0 mg/eye STK-002 treatment groups. At 4 weeks after a single injection of STK-002, detectable nonproductive RNA transcripts in the retinal samples were reduced to ∼53% (0.1 mg/eye), 12% (0.3 mg/eye), and 6% (1.0 mg/eye) when compared with the Vehicle group (Fig. 5A). Additional 0.3 and 1.0 mg/eye treatment groups were also evaluated at 8 weeks and found to maintain similar reductions. We observed a corresponding increase in retinal OPA1 protein quantified by ELISA. At week 4, the level of increase was 31%, 47%, and 44% in the retinal tissue at STK-002 doses of 0.1, 0.3, and 1.0 mg/eye, respectively (Fig. 5B). The 0.3 and 1.0 mg dose levels were also assessed in an additional cohort at week 8, and elevated OPA1 protein levels were maintained. Percent increase could not be determined since there were no control animals at week 8; however, the OPA1 level measured by ELISA was comparable and slightly higher than the respective week 4 dose group levels (Fig. 5B). These data indicate that a single IVT administration of STK-002 was effective for at least 8 weeks in significantly reducing the levels of nonproductive mRNA transcripts and increasing OPA1 protein in the cynomolgus monkey retina.

Retinal tissue levels of STK-002 were quantitated at weeks 4 and 8. The exposure was dose dependent but not strictly dose proportional in retinal tissue (Fig. 5C). At week 8 compared with week 4, mean STK-002 concentration in the retina decreased by 34% in the 0.3 mg/eye group and remained at a similar level for 1 mg/eye dose (increased by 6%) (Fig. 5C). This sustained retention through week 8 suggests slow clearance and long tissue half-life of STK-002 in retina. Furthermore, clinical ophthalmic exams showed that STK-002 was well tolerated at all dose levels tested for the duration of the study.

### STK-002 and OPA1 protein are detected in cynomolgus monkey RGCs after IVT administration

The loss of visual function in ADOA is due to the dysfunction and loss of RGCs, thus therapeutic targeting of this cell type will be critical for successful treatment of ADOA. Sections of Modified Davidson’s-fixed paraffin-embedded cynomolgus monkey retina from the Vehicle and 0.1, 0.3, and 1.0 mg/eye STK-002 treatment groups were evaluated by miRNAscope *in situ* hybridization to detect STK-002. PBS-injected eyes did not demonstrate any detectable signal of STK-002, indicating that the probe was highly specific (Fig. 5D). By visual inspection as well as quantitative histological assessment (Supplementary Fig. S14) we observed a dose-related increase in STK-002 signal in the RGC layer at week 4.

By visual inspection after antibody staining, OPA1 protein immunofluorescence was observed in the RGC layer of the retina. The intensity of immunofluorescence appeared to increase with STK-002 dose in the RGC cell layer, including RGCs in the foveal region (Fig. 5E) at 4 weeks after STK-002 treatment. However, results of attempted quantitation of the immunofluorescence intensity showed an increasing trend but were not statistically significantly different from Vehicle (Supplementary Fig. S15). As expected and consistent with published literature,^23^ a substantial signal for OPA1 protein was apparent in the RGCs at baseline (Fig. 5E, PBS).

## Discussion

TANGO, the technology exemplified by STK-002, uses ASO modulation of pre-mRNA splicing to prevent the synthesis of naturally occurring nonproductive mRNA to increase protein levels, thereby restoring the target protein toward normal levels in the case of haploinsufficiency.^15^ ADOA due to *OPA1* haploinsufficiency is highly suited to benefit from this technology. Importantly, the *OPA1* gene includes a targetable NMD event that we have verified to exist in the human retina. Most patients with ADOA have a mutation of one allele of *OPA1* that results in a null allele,^5^ leaving one WT *OPA1* allele that can be leveraged by STK-002.

An important aspect of STK-002-mediated OPA1 augmentation is that it is agnostic to the specific loss-of-function mutation on the affected allele, unlike other approaches recently reported. These include a U1 snRNA-adapted strategy to silence exon skipping on the mutant allele,^24^ and a CRISPR-Cas9-guided homology-directed repair of an *OPA1* mutation.^25^ These approaches will likely require the development of variant-specific therapies, reducing their applicability. Augmentation of OPA1 through AAV gene expression is another therapeutic modality currently being explored; however, the size limitation for AAV cargo means that only a single OPA1 isoform can be expressed.^26^ A further hypothetical advantage of STK-002 is the ability to increase expression of multiple OPA1 isoforms, although this needs to be confirmed by detailed isoform analysis.

Several lines of evidence exist indicating that individuals with haploinsufficiency, with reduced levels of cellular OPA1 protein compared with WT individuals, can benefit from OPA1 augmentation. Cells derived from patients with ADOA, including lymphoid cells and fibroblasts, demonstrate consistently reduced mitochondrial function, with variably abnormal mitochondrial morphology and increased susceptibility to apoptosis.^6,12,13,27^ Augmentation of OPA1 in patient cells, as shown by others using AAV expression,^28^ CRISPR-Cas9-directed *OPA1* mutation repair^25^ or by STK-002 as shown in this work, can improve parameters of mitochondrial respiratory function. Interestingly, similar to our results, Maloney et al.^28^ observe that partially rescued OPA1 levels were beneficial to mitochondrial function. Furthermore, the ability of STK-002 to enhance the ability of cells to respond to mitochondrial stress is mutation independent, which supports the possibility to develop a single treatment for all ADOA haploinsufficient patients.

One limitation of our approach is that the increase in OPA1 expression may potentially be limited to the abundance of the nonproductive transcript in a particular cell type. Even though the nonproductive transcript is detectable in multiple human retinal tissue samples, we are unable to estimate its true abundance given that NMD is active and ongoing in the tissue. This precludes us from determining how much OPA1 protein increase can potentially be achieved in human retina. Interestingly, the average nonproductive transcript abundance between human and monkey retina is very similar (3.8% in human retina and 3.57% in monkey retina) and therefore, one may speculate a similar extent of OPA1 protein response in human retina as seen in the monkey (up to 47% increase, Fig. 5B).

Rodent models have been engineered with heterozygous *Opa1* mutations resulting in haploinsufficiency that recapitulate features of ADOA, including RGC death and visual function abnormality.^29–31^ Importantly, OPA1 augmentation in these animals with IVT AAV vectors can reduce or prevent further RGC loss and protect visual function as assessed by electrophysiological testing.^32^ The NMD-inducing exon targeted by STK-002 is not conserved in rodents, and thus we were not able to make use of these mouse models. However, since the mechanism of action of STK-002 utilizes the WT allele, we performed *in vivo* studies in the WT rabbit (with a rabbit-specific surrogate ASO) and cynomolgus monkey. These studies limited us to the demonstration of a pharmacodynamic rather than a therapeutic effect. Nevertheless, we observed substantial and sustained OPA1 increase in the retina of both rabbit and monkey. Furthermore, we established that STK-002 is taken up by RGCs of STK-002-treated monkeys.

ASOs delivered intravitreally have been shown to distribute broadly through the retina,^33,34^ and as we have shown, STK-002 is taken up by RGCs, the target cells for ADOA. Additionally, data from others^35^ and data presented in this study indicate sustained retinal tissue exposure and pharmacology after a single IVT injection of ASO, supporting the possibility of infrequent administration in the clinic. ASOs in general^36,37^ have shown a promising safety profile in the eye, and clinical ophthalmic exams performed in both the monkey and rabbit studies showed that both STK-002 and its rabbit surrogate were well tolerated at dose levels that mediated robust pharmacology in the retina. Taken together, these data support the continued development of STK-002, a targeted and potentially disease-modifying therapy for ADOA, which is currently untreatable. Preclinical and toxicology studies are currently ongoing to establish the safety profile for STK-002 to support its entry into Phase 1 clinical studies in patients with confirmed genetic diagnosis of ADOA caused by *OPA1* mutations.
