# Supplementary material for: Antisense Oligonucleotide STK-002 Increases OPA1 in Retina and Improves Mitochondrial Function in Autosomal Dominant Optic Atrophy Cells
Source: Nucleic Acid Ther. 2024 Oct 16;34(5):221–33. doi: 10.1089/nat.2024.0022 (PMC11564677; doi:10.1089/nat.2024.0022)
Supplement: Supplementary Figure S1 [file nat.2024.0022_supplementary_data.pdf]

Supplemental Materials

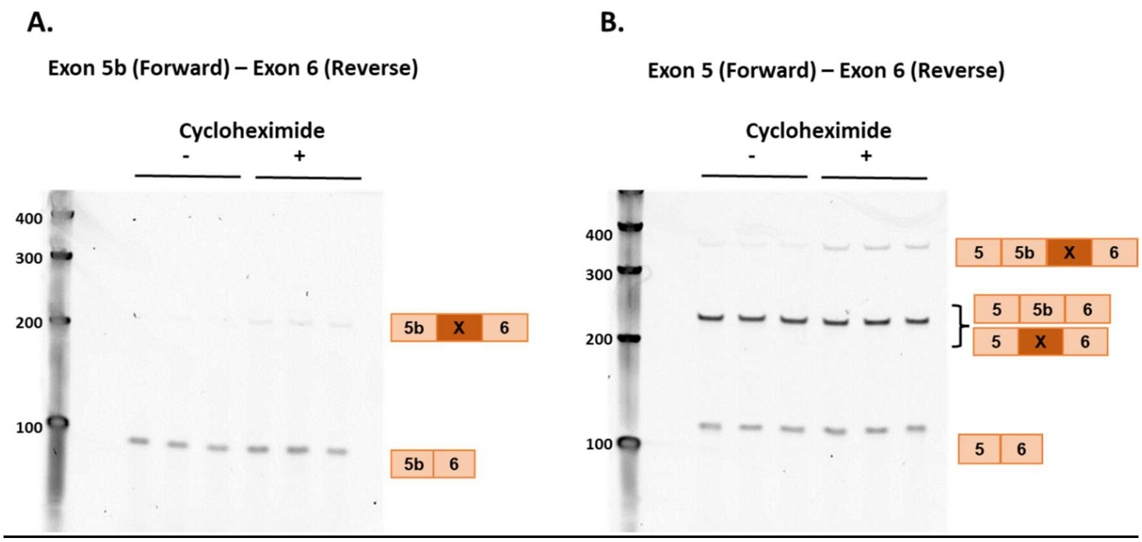

Figure S1. Validation of the nonproductive splicing event in *OPA1*. Refer Fig. 1A for schematic of alternative splicing around exons 5, 5b, X and 6. RT-PCR assays were designed with reverse primers on exon 6 and forward primers on exon 5b (A) or exon 5 (B). Treatment of HEK293 cells with CHX for 3 h (50µg/mL final concentration) increases the abundance of nonproductive transcripts containing Exon X. The middle band in (B) potentially includes both 5-5b-6 and 5-X-6 transcripts due to similar size of exons 5b and X (111bp versus 108bp respectively). This complex alternative splicing makes it difficult to calculate Exon X abundance accurately by RT-PCR.

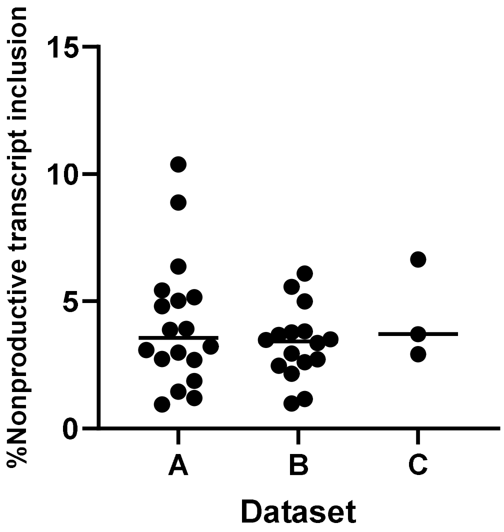

<u>Figure S2. Nonproductive transcript abundance in human retina.</u> Transcripts Per Million (TPM) values were obtained from RNA-sequencing datasets ERP126780 (A), SRP015336 (B) and SRP107937 (C). In the ERP126780 dataset, 32 samples with low read coverage and extreme 3' bias were excluded. The percentage of nonproductive transcript inclusion in these samples was calculated as [nonproductive transcript TPM] / [total OPA1 transcripts TPM] and expressed as a percentage. The average abundance across these samples was 3.8%.

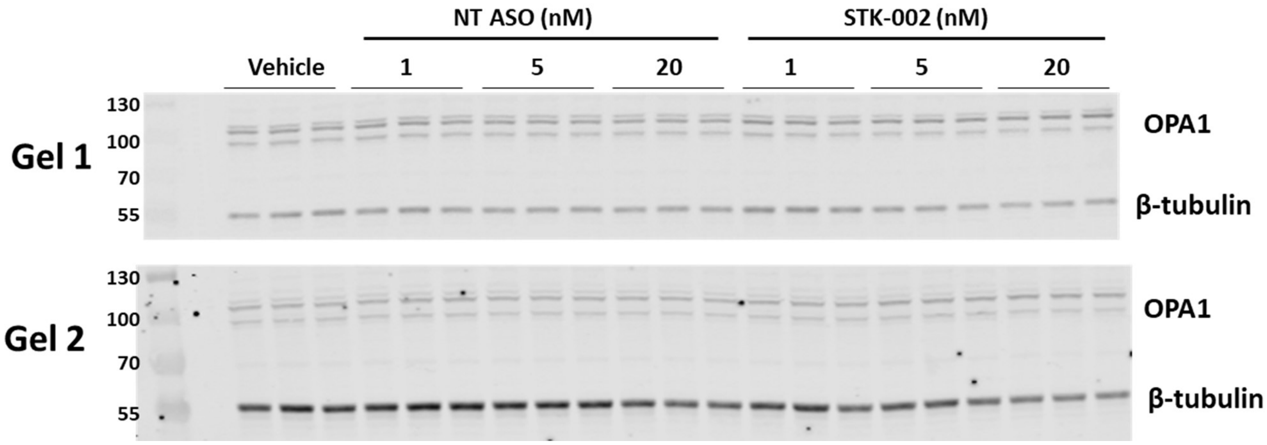

Figure S3. Western blot image for Figure 1C. HEK293 cells were transfected with Vehicle or 1, 5, 20nM doses of STK-002 or non-targeting (NT) ASO and effect on OPA1 protein was evaluated after 72 h by western blot. Each lane on each gel represents one biological replicate. Quantification is presented in Fig. 1E. Protein ladder molecular weights (kDa) are included.

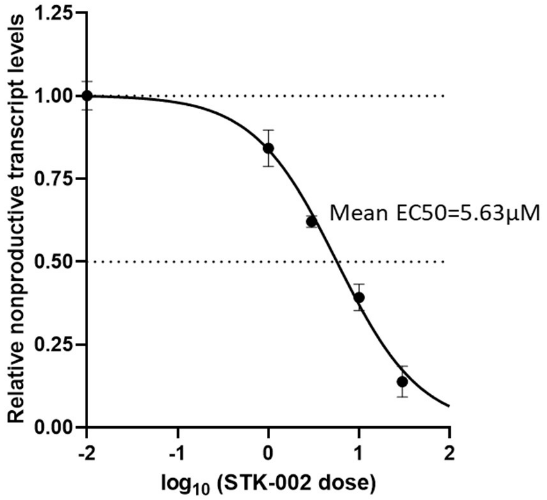

Figure S4. EC50 evaluation of STK-002 in HEK293 cells. HEK293 cells were treated with 1, 3, 10 and 30 µM doses of STK-002 or Vehicle for 72 h by gymnotic uptake. Media with CHX (50 µg/mL final concentration) was applied for the last 3 h of treatment. The relative abundance of the *OPA1* nonproductive transcript was determined using a qPCR primer probe assay normalized to house-keeping gene RPL32 and plotted against the (log10) of the ASO dose using a nonlinear regression of four parameter dose-response curve fit. A representative dose-response curve fit is shown above. Plotted values represent mean of at least two biological replicates; error bars represent S.D. Mean EC50 for STK-002 was averaged over ten independent experiments performed on different days and calculated as 5.63 µM.

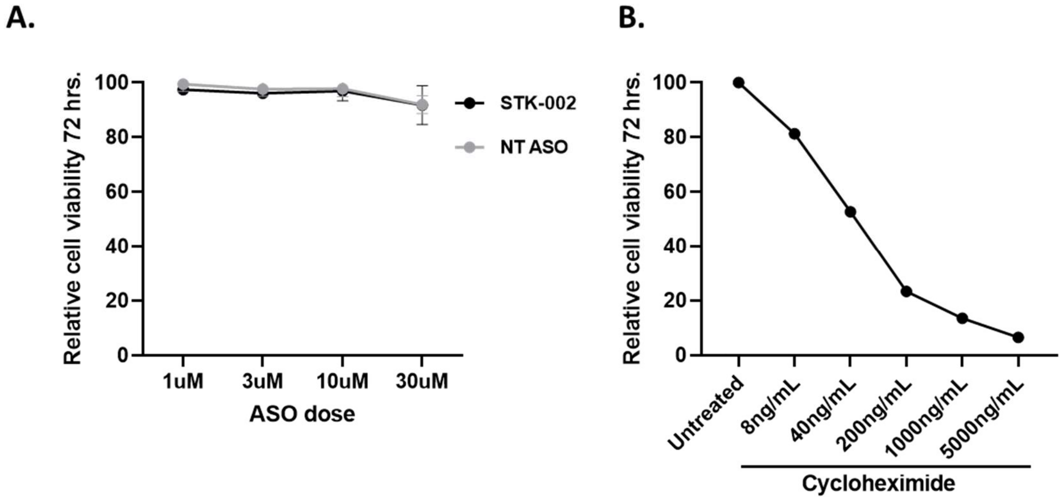

Figure S5. STK-002 does not affect HEK293 cell viability. (A) HEK293 cells were treated with 1, 3, 10 and 30 µM doses of STK-002 or NT ASO for 72 h by gymnotic uptake and cell viability was determined using the RealTime-Glo MT Cell Viability Assay. Luminescence values were normalized to cells treated with a vehicle (water) control. The mean ± SD of three biological replicates is shown. (B) As a positive control for the assay, HEK293 cells were treated with different concentrations of CHX for 72 h and a dose-dependent reduction in cell viability was observed.

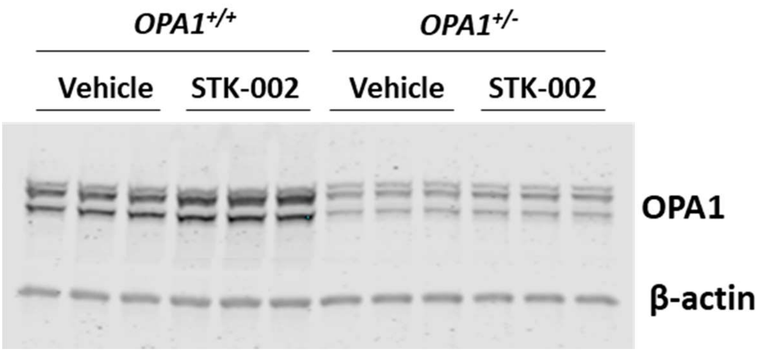

Figure S6. Western blot image for Figure 2A. *OPA1*$^{+/+}$ and *OPA1*$^{+/-}$ HEK293 cells were treated with Vehicle or 10µM STK-002 by gymnotic uptake and assessed for effect on OPA1 protein 72 h

post treatment by western blot. Each lane represents one biological replicate. Quantification is presented in Fig. 2A.

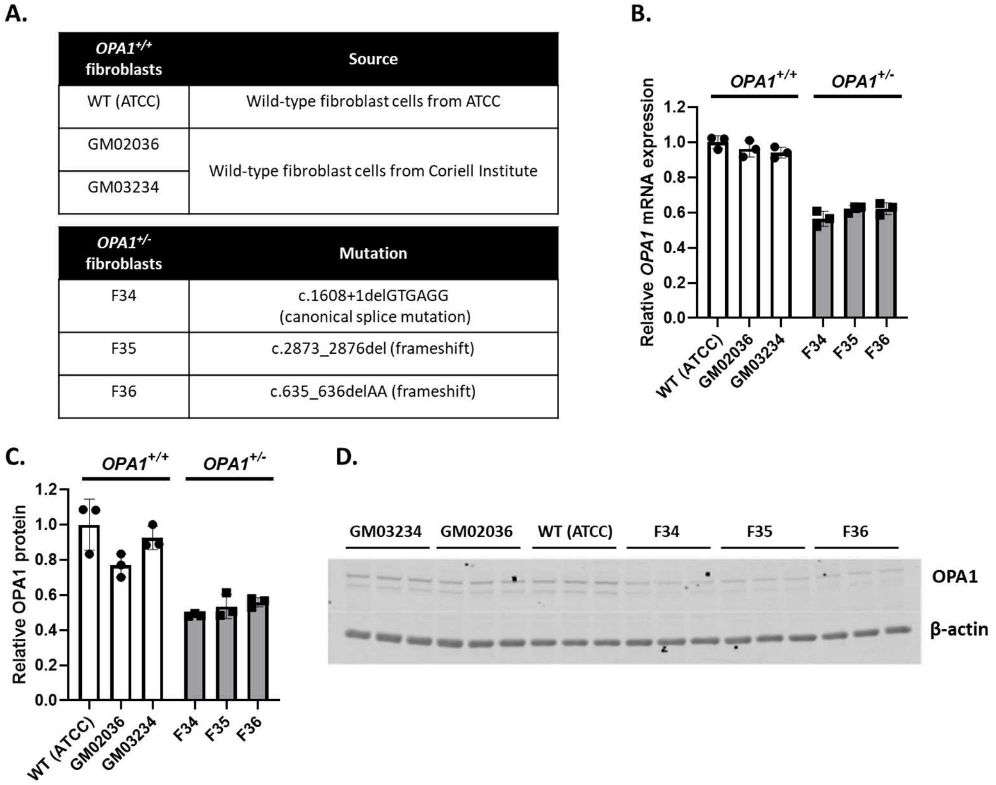

Figure S7. Comparison of OPA1 expression and nonproductive transcript abundance in ADOA patient fibroblasts to three different WT primary fibroblast cells. (A) Information on the three primary WT fibroblast cells evaluated and *OPA1* variant information for the three ADOA patients designated as F34, F35 and F36. (B) qPCR analysis of total *OPA1* mRNA levels in the six fibroblast cells normalized to *RPL32*. (C, D) OPA1 protein evaluation normalized to β-actin. Data in (B) and (C) are expressed relative to WT fibroblasts from ATCC.

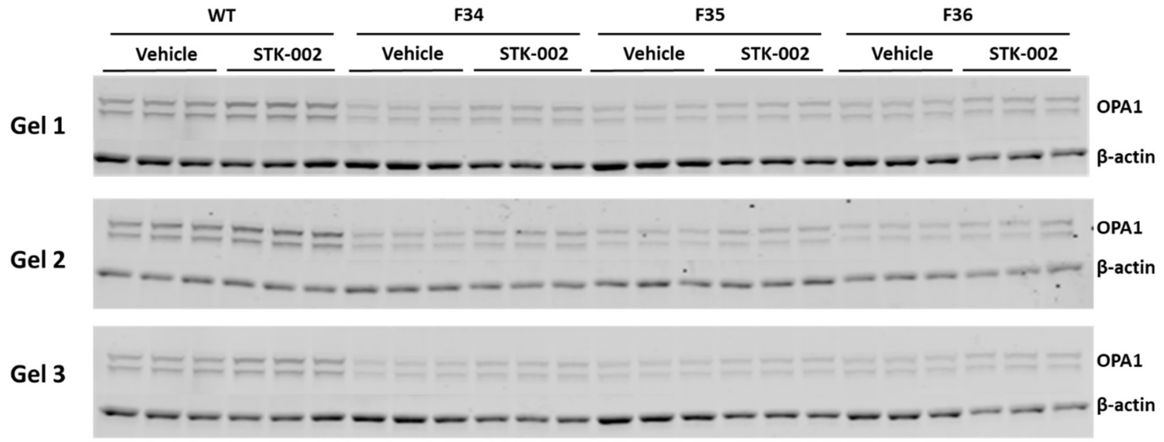

Figure S8. Western blot images for Figure 3B. WT (ATCC fibroblasts) and all three patient fibroblast cells were treated with 40nM STK-002 by transfection and analyzed after 72 h for effect on OPA1 protein normalized to β-actin. Each lane of each gel represents one biological replicate. Quantification presented in Fig. 3B.

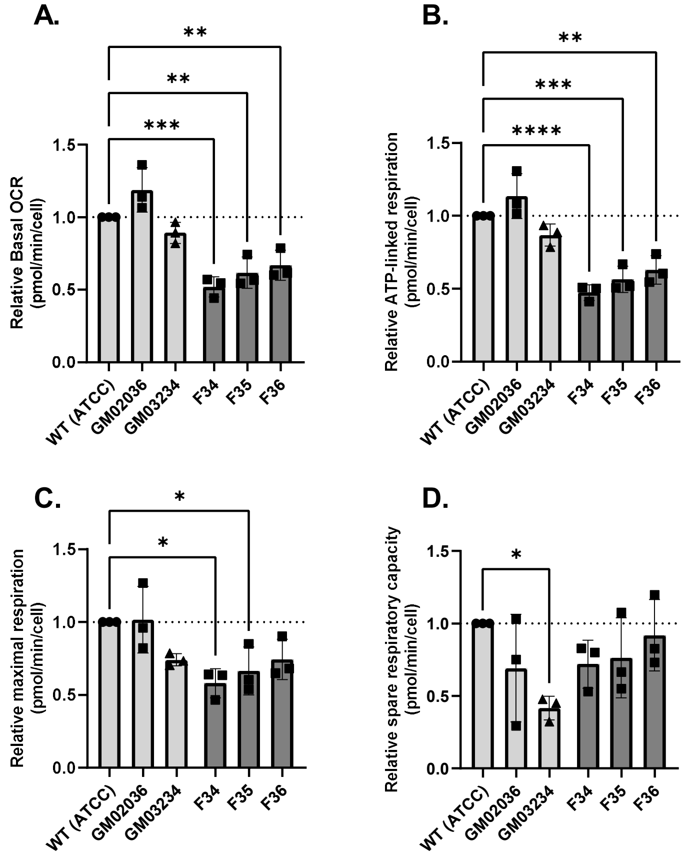

Figure S9. Reduced mitochondrial function in ADOA patient derived fibroblast cells.

Mitochondrial function in three WT and ADOA patient derived fibroblast cells was assessed by measuring oxygen consumption rate (OCR; pmol/min/cell) under both basal and stress conditions in a Seahorse XFe96 extracellular flux analyzer and normalized to cell count. Data represent mean ± SD of three biological replicates and plotted relative to WT cells from ATCC. Each data point represents an average of OCR values from 8-12 wells. *P<0.05; **P<0.01; ***P<0.001; and ****P<0.0001 by ordinary one-way ANOVA followed by Dunnett's multiple comparison test relative to WT cells from ATCC.

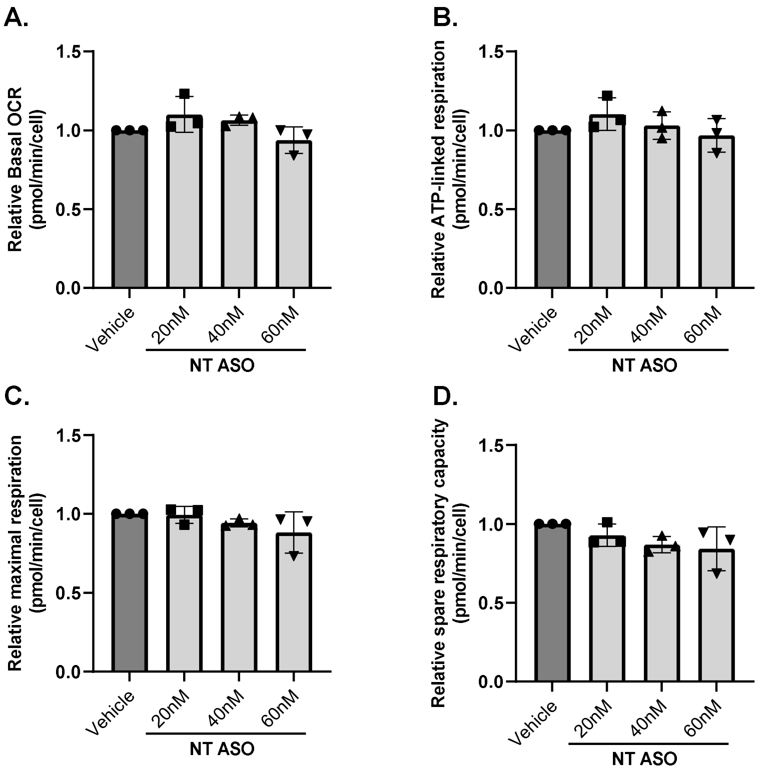

Figure S10. Non-targeting ASO does not change OCR parameters in F35 cells. F35 patient fibroblast cells were treated with 20, 40 or 60nM NT ASO for 72 h and OCR (pmol/min/cell) under both basal and stress conditions was measured by a Seahorse XFe96 extracellular flux analyzer and normalized to cell count as performed in Figure 5. Data represent mean ± SD of three biological replicates and plotted relative to the Vehicle group for each parameter. Each data point represents an average of OCR values from 8-12 wells.

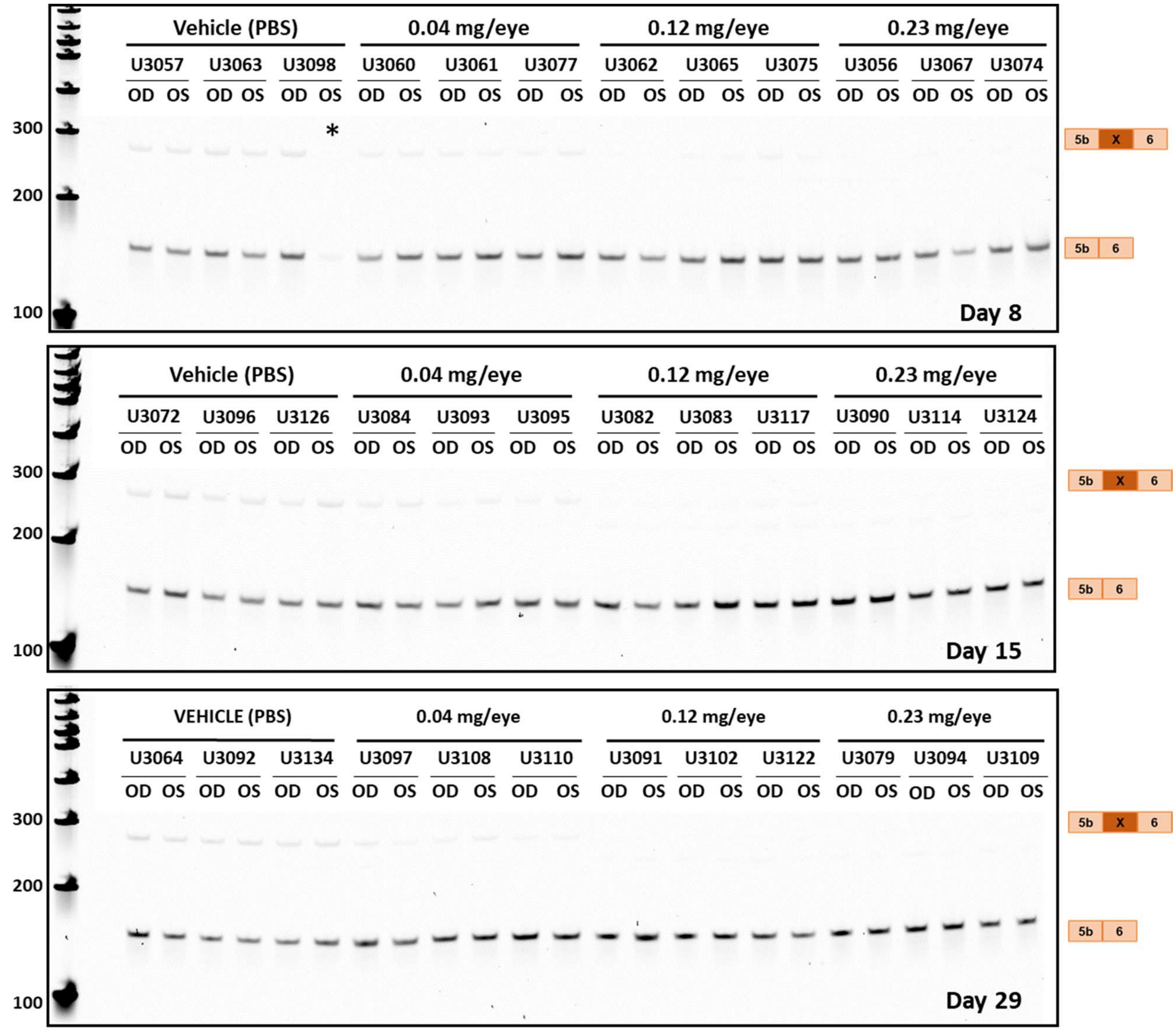

Figure S11. RT-PCR gel images for Fig. 4B. * Sample excluded from analysis. DNA ladder molecular weights (bp) are indicated.

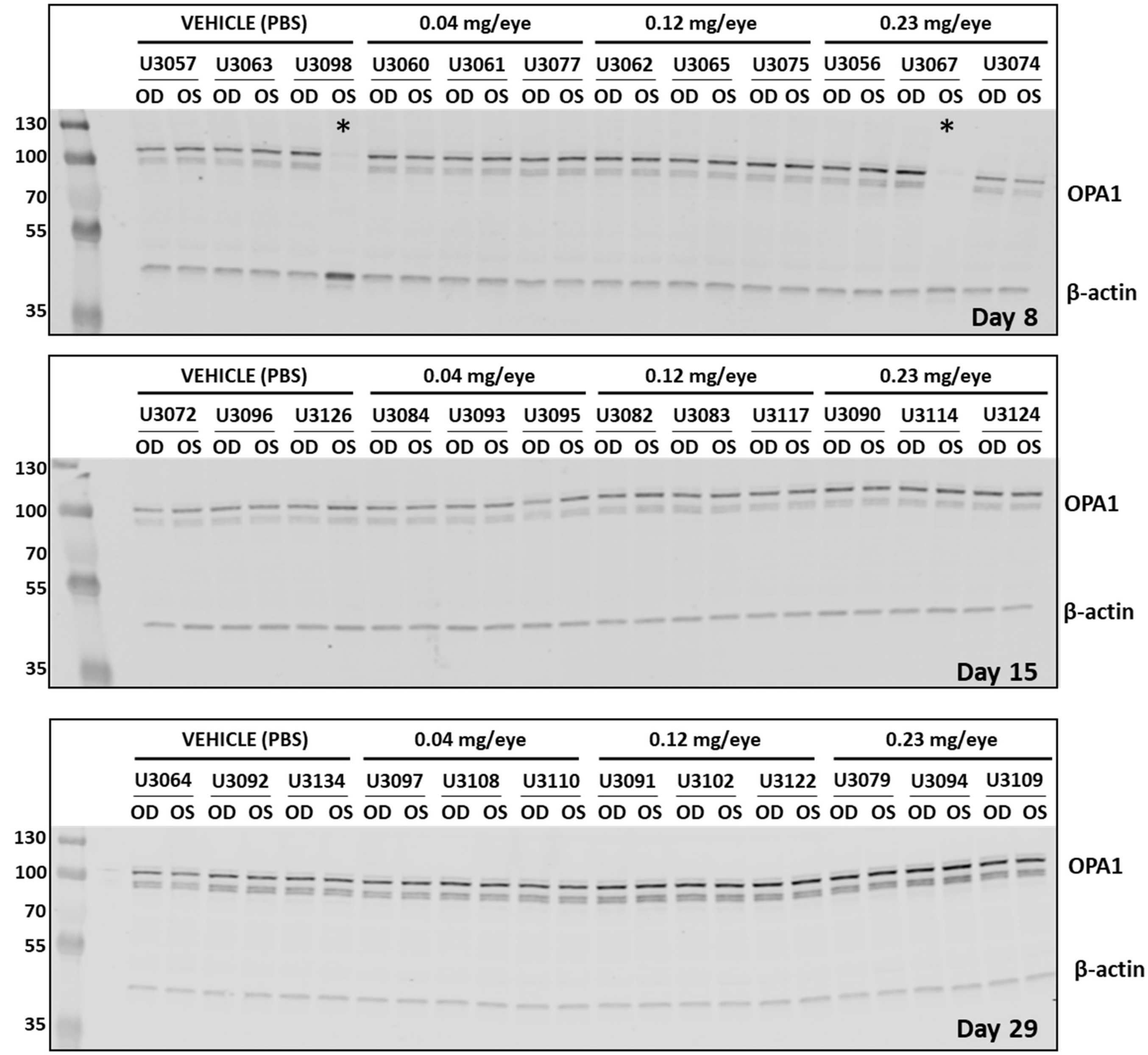

Figure S12. Western blot images for Fig. 4C. * Samples excluded from analysis. Protein ladder molecular weights (kDa) are indicated.

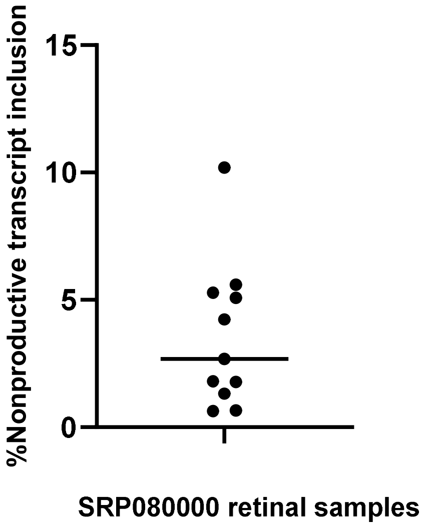

Figure S13__. Nonproductive transcript abundance in cynomolgus monkey retina.__ Transcripts Per Million (TPM) values were obtained from the 11 retinal samples in the RNA-sequencing dataset SRP080000. The percentage of nonproductive transcript inclusion in these samples was calculated as [nonproductive transcript TPM] / [total OPA1 transcripts TPM] and expressed as a percentage. The average abundance across these samples was 3.57%.

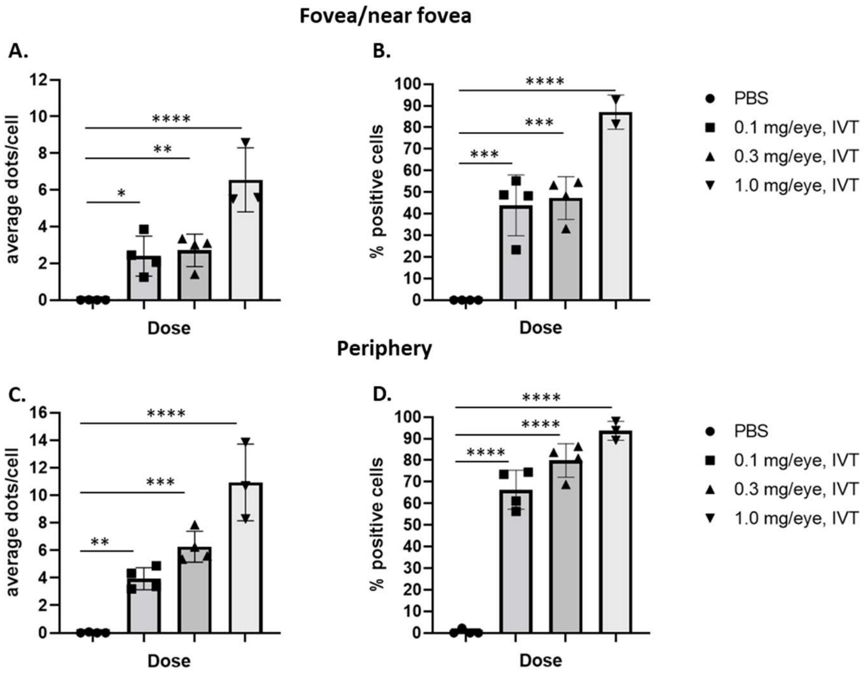

Figure S14. Dose-related increase in STK-002 is detected in RGCs after intravitreal (IVT) administration to cynomolgus monkeys. Quantitation of mRNA copies (dots) per cell and percent positive RGCs in foveal (A, B) and peripheral retina (C, D) showed a dose-related increase. Statistics by ordinary one-way ANOVA with Dunnett's multiple comparison test. *P<0.05, **P<0.01, ***P<0.001, ****P<0.0001 compared to Vehicle.

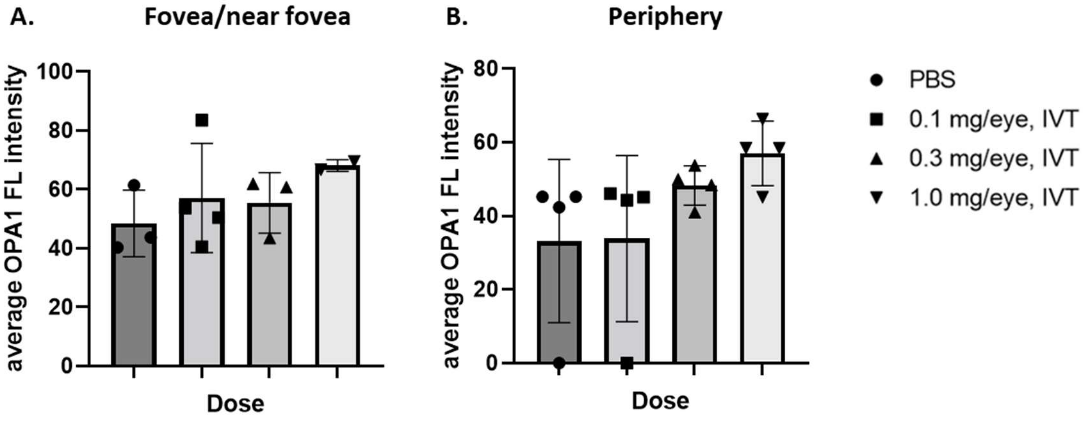

Figure S15. Quantitation of OPA1 protein immunofluorescence in cynomolgus monkey RGCs after intravitreal STK-002 administration. At 4 weeks after intravitreal administration of STK-002, immunofluorescent staining of OPA1 protein was performed on sections from MDF-fixed, paraffin-embedded eyes from indicated treatment groups. Fluorescence (FL) intensity was quantitated in the RGC layer in foveal/near foveal (A) and peripheral (B) retina, however only a trend towards dose-associated increase was observed that was not statistically significant. Note substantial OPA1 signal in RGCs at baseline (Vehicle). Each retina analyzed is represented by a single data point on the graph.

Supplemental Tables:

**Primer**	**Sequence**
Human *OPA1* forward primer (exon 5b)	5’-GCCAATATAGCACGAGCTAT-3’
Human *OPA1* forward primer (exon 5)	5’-AAGAAACGGCGTTTAGAGCAA-3’
Human *OPA1* reverse primer (exon 6)	5’-CTGAGTGTGCAGAAGTTCTTCC-3’
Rabbit *OPA1* forward primer (exon 5b)	5’-TGCTTGGTGAGCTCATTCTC-3’
Rabbit *OPA1* reverse primer (exon 6)	5’-GAAGTTCTTCTTGAAGTTGGTCA-3’

Table S1. RT-PCR primer information.

**Temperature and time**	**Cycles**
95°C for 9 min	1X
• 95°C for 30 sec	28X

• 52°C (human), 54°C (rabbit) for 30 sec<br>• 72°C for 30 sec	
72°C for 10 min	1X
4°C	hold

Table S2. Cycle conditions for exon 5b (forward) – exon 6 (reverse) RT-PCR

Temperature and time	Cycles
95°C for 9 min	1X
• 95°C for 30 sec<br>• 55°C (human) for 30 sec<br>• 72°C for 30 sec	26X
72°C for 10 min	1X
4°C	hold

Table S3. Cycle conditions for exon 5 (forward) – exon 6 (reverse) RT-PCR

qPCR assay information. Probe-based qPCR assays were either purchased from Thermo Fisher or custom designed by IDT. qPCR was performed with the following cycle conditions: 50°C for 2 min, 95°C for 10 min, followed by 40 cycles (95°C for 15 sec, 60°C for 1 min). The commercially purchased assays were as follows: human *OPA1*: Hs01047013_m1, human *RPL32*: Hs00851655_g1, cyno β-actin: Mf04354341_g1.

Sequences of the custom-designed qPCR assays were as follows:

Primer/probe	Sequence (5' to 3')

Forward Primer	ATTCCGCTCTTTCTCCATCAG
Reverse Primer	CTCTTTGTCTGACACCCTGAC
Probe	/56-FAM/AG CTC TGG T/Zen/TTCAAGACCATTGATATCC A/3IABkFQ/

Table S4. qPCR assay for detection of human nonproductive *OPA1* transcript.

**Primer/Probe**	**Sequence**
Forward Primer	5′-AGC TCT GGT TTC AAG ACC ATT-3′
Reverse primer	5′-GCA GAA GTT CTT CTT GAA GTT GG-3′
Probe	5′-/56-FAM/CC ATT TCT G/Zen/T CAG GGT GTC AGA CAA AGA /3IABkFQ/-3′

Table S5. qPCR assay for detection of cyno nonproductive *OPA1* transcript.
